# Obstetric Hemorrhage Outcomes by Intrapartum Risk Stratification at a Single Tertiary Care Center

**DOI:** 10.7759/cureus.6456

**Published:** 2019-12-24

**Authors:** S Ahmed Hussain, Carla B Guarini, Colleen Blosser, Aaron T Poole

**Affiliations:** 1 Obstetrics and Gynecology, Naval Medical Center Portsmouth, Portsmouth, USA; 2 Obstetrics and Gynecology, Women's Health, Naval Medical Center Portsmouth, Portsmouth, USA; 3 Obstetrics and Gynecology, Maternal-Fetal Medicine, Naval Medical Center Portsmouth, Portsmouth, USA

**Keywords:** postpartum hemorrhage, blood transfusion, risk stratification

## Abstract

Introduction

Postpartum hemorrhage is a leading cause of maternal mortality worldwide. Performance of a postpartum hemorrhage risk assessment prior to delivery has been recommended to identify patients at higher risk for hemorrhage to support advanced planning for optimal response. The objective of this quality improvement initiative is to evaluate the transfusion and hemorrhage rates for patients at low, moderate, and high risk for postpartum hemorrhage by utilizing standardized risk assessment.

Methods and materials

A historic cohort study was performed among women delivering from March 2017 to June 2018 at a single United States military tertiary medical center. A postpartum hemorrhage risk assessment was performed utilizing the California Maternal Quality Care Collaborative toolkit for all patients admitted to Labor and Delivery and when the postpartum hemorrhage risk increased during the intrapartum period. An electronic log was reviewed to determine blood loss volume, change in hematocrit, and transfusion rates in patients at low, moderate, and high risk for postpartum hemorrhage for all deliveries, stratified by delivery type.

Results

There were 3,377 deliveries during the study period with 145 excluded due to lack of assigned risk category. The high-risk group (12.3% of deliveries) was 4.3 times more likely to receive a blood transfusion, 2.9 times more likely to have a blood loss over 1000 mL, and 2.1 times more likely to have a transfusion or hematocrit drop of 10 points when compared with the low-risk group (69.4% of deliveries). Of those transfused, the majority were classified as low risk as this was the most common assignment.

Conclusion

Risk stratification can differentiate low from high-risk patients for postpartum hemorrhage and associated transfusion or change in hematocrit. However, the majority of patients who receive a transfusion will be classified as low or moderate risk. Thus, all patients should be monitored closely and treated aggressively to prevent morbidity.

## Introduction

Postpartum hemorrhage (PPH) remains the leading cause of maternal mortality worldwide, affecting up to 5% of deliveries and leading to 1.7 deaths per 100,000 live births, or 86,000 maternal deaths annually worldwide [1-3]. Postpartum hemorrhage is most commonly defined as blood loss greater than or equal to 1,000 mL or blood loss accompanied by signs or symptoms of hypovolemia within 24 hours after delivery. Though risk factors have been identified, accurate prediction of postpartum hemorrhage remains elusive [2].

Recognition of postpartum hemorrhage is imprecise due to errors in estimation of blood loss by delivering providers [4]. Providers tend to underestimate blood loss by 30% [5]. Concurrent with this adjustment to the Labor and Delivery order bundle, our hospital also implemented quantitative blood loss (QBL) as a method for estimating the blood loss in each delivery. Prior to the introduction of QBL, blood loss from each delivery was estimated by the delivering provider. Calculation of QBL involves measuring blood loss with a calibrated drape as well as obtaining the weights of other blood collection tools such as laparotomy sponges. The implementation of QBL can improve the accuracy of blood loss estimation to within 15% of actual blood loss [6].

In 2010, the California Maternal Quality Care Collaborative (CMQCC) released a toolkit designed to improve management and response to PPH. This toolkit included a postpartum hemorrhage risk assessment at the time of admission [3,7]. Assessment of factors known to increase the likelihood of PPH allowed stratification of each patient into either low, medium or high-risk categories, which then determined the need for either a blood type and antibody screen for low or medium risk patients or a blood type and cross-match for high-risk patients. It was demonstrated that implementation of this early risk assessment allowed for accurate identification of patients likely to experience PPH, require blood transfusion, require additional procedures following delivery, require ICU admission, or have an extended inpatient stay [3].

The present study aims to determine the clinical utility of a similar postpartum hemorrhage risk stratification method to identify patients at high risk of life-threatening bleeding after delivery at our facility as a quality improvement initiative which included the re-categorization of patients in labor at each change of shift.

## Materials and methods

Prior to the initiation of this retrospective cohort study, the clinical investigation department at Naval Medical Center Portsmouth (NMCP) deemed the study IRB exempt as quality improvement research. Beginning March 2017, all patients admitted to Labor and Delivery at NMCP were assigned a postpartum hemorrhage risk stratification based on the CMQCC tool kit and American College of Obstetricians and Gynecologists (ACOG) recommendations [2, 7-9]. While the CMQCC excluded estimated fetal weight over 4000 grams or body mass index over 40, ACOG recommended including these risk factors. The ACOG factors considered in risk stratification included BMI ≥ 40, prior cesarean section, multiple gestation, multiparity (defined as more than four prior births), history of PPH, large uterine fibroids, fetal macrosomia, polyhydramnios, hematocrit less than 30%, prolonged oxytocin exposure, prolonged second stage of labor, magnesium sulfate infusion, placenta previa or low lying placenta, accreta spectrum, low platelet count, active bleeding, and known coagulopathies (Table 1) [3]. A type and screen was performed for all patients meeting criteria for low or moderate PPH risk, while a type and cross-match for two units of packed red blood cells was performed for all patients meeting criteria for high PPH risk. The type and screen was upgraded to a type and cross for all patients who meet criteria intrapartum for high PPH risk if they were deemed to be low or moderate risk on admission with re-evaluation of risk stratification at least every 12 hours.

**Table 1:** Postpartum hemorrhage risk stratification criteria Based upon American College of Obstetrics and Gynecology guidelines and California Maternal Quality Care Collaborative (CMQCC) [3-6]. EFW: Estimated fetal weight; BMI: Body mass index; PPH: Postpartum hemorrhage.

Low Risk Type and Screen	Medium Risk Type and Screen	High Risk Type and Cross
Admission Factors		
No previous uterine surgery	Prior cesarean, uterine surgery, or multiple laparotomies	Two or more medium risk factors
Singleton pregnancy	Multiple gestation	Placenta previa/low lying
≤4 previous births	>4 prior births	Suspected accreta spectrum
EFW < 4000 g	History of PPH	Platelets < 70,000
BMI < 40	Large Myomas (>5 cm)	Active bleeding
No bleeding disorder	EFW ≥ 4000 g	Known coagulopathy
No history of PPH	BMI ≥ 40	
	Hematocrit < 30%	
	Polyhydramnios	
Intrapartum Factors		
No prolonged labor course	Suspected Triple I	New active bleeding
	Prolonged oxytocin > 24 hr	
	Prolonged 2^nd^ stage greater than 3 hours	
	Magnesium Sulfate	

A report was generated from our hospital’s electronic medical record containing all deliveries between March 1, 2017 and June 1, 2018. This report included each patient’s PPH risk determination, mode of delivery, QBL, need for blood transfusion, and hematocrit decrease after delivery. Routine assessment of hematocrit is not performed on vaginal deliveries without signs or symptoms of postpartum hemorrhage. All cesarean deliveries have hematocrit drawn at 8 hours postpartum. Postpartum hemorrhage was defined as a QBL of greater than or equal to 1,000 mL with determination of frequency of blood transfusion and hematocrit difference of greater than or equal to 10% between their antepartum and postpartum values.

Statistical analyses were performed using GraphPad Prism 7 software (release 7; GraphPad Software, La Jolla, CA). Kruskal-Wallis, Fisher’s exact and Chi-squared tests for trend were used as appropriate. A two-sided probability of <.05 was accepted as statistically significant.

## Results

**Figure 1:**
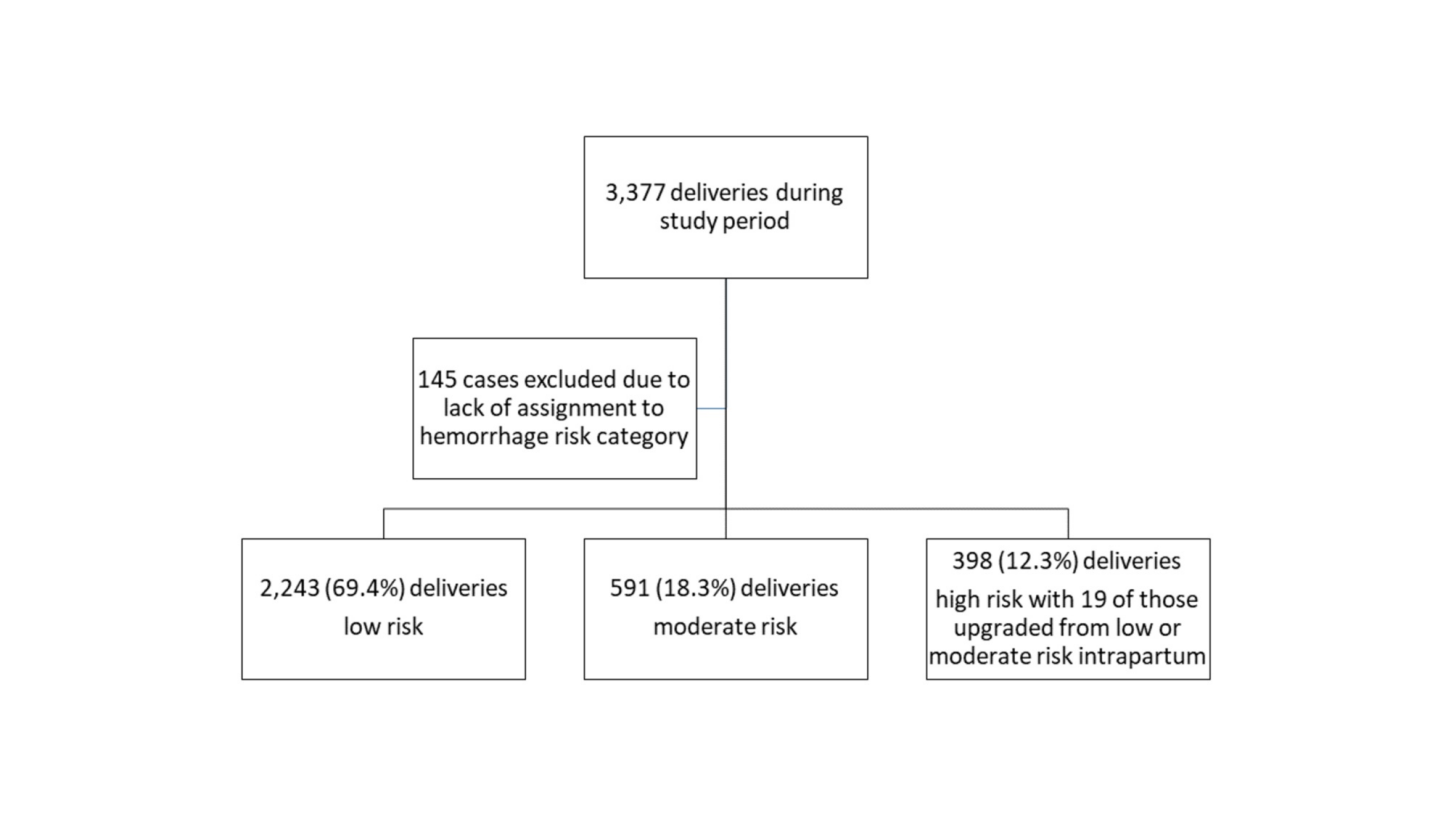
Flow sheet for obstetric risk stratification

There were 3,377 patients who delivered during the study period with 70 (2.1%) who received a blood transfusion. One hundred forty-five cases were excluded due to lack of assignment to a risk category, resulting in 3,232 deliveries with an assigned postpartum hemorrhage risk. Of these, 2243 (69.4%) were deemed low risk at delivery, 591 (18.3%) moderate risk, and 398 (12.3%) high risk. Of the high-risk patients, 19 (4.8%) were upgraded to this stratification during their intrapartum course (Figure 1).

The mean quantitative blood loss was different between each group with 350 mL in the low PPH risk group, 493 mL in the moderate PPH risk group, and 561 mL in the high PPH risk group (P < .001, Table 2).

**Table 2:** Hemorrhage outcomes by risk stratification All P-values < .001. Chi-squared test for trend for categorical variables and Kruskal-Wallace for continuous data were used. QBL: Quantitative blood loss

	Low		Moderate		High	
Total Deliveries	2243	69.4%	591	18.3%	398	12.3%
Cesarean Deliveries	466	20.8%	291	49.2%	226	56.8%
Mean QBL (mL)	350		493		561	
Postpartum Hemorrhage	84	3.7%	46	7.8%	44	11.1%
Transfusions Received	30	1.3%	12	2.0%	23	5.8%

There was a 2.7 times higher rate of cesarean delivery in the high-risk group compared to low risk while there was a 2.4 times higher rate in the moderate risk group compared to low risk. The rate of blood transfusion among those with cesarean delivery was highest among those in high-risk stratification verse medium or low risk (6.6% high, 3.1% medium, 3.6% low, P < .001) with an odds ratio of 1.92 (95% CI: 1.02-3.58, P = .04) of being transfused in high-risk group compared to medium or low risk. Similarly, the rate was elevated among vaginal deliveries deemed high risk (4.7% high, 1.0% medium, 0.7% low, P < .001) with an odds ratio of 6.7 (95% CI: 2.95-15.4, P < .001) for transfusion of high-risk patients vs. medium or low risk.

Across all risk categories there were 174 (5.4%) postpartum hemorrhages, defined as a QBL greater than or equal to 1,000 mL. Among the low-risk patients, there were 84 postpartum hemorrhages, representing 3.7% of low-risk deliveries. Among the moderate risk patients, there were 46 postpartum hemorrhages, representing 7.8% of moderate risk deliveries. Among the high-risk patients, there were 44 postpartum hemorrhages, representing 11.1% of high-risk deliveries. Patients assigned the high PPH risk stratification were 2.9 times more likely to have a postpartum hemorrhage than patients assigned a low PPH risk and 2.5 times more likely to have a postpartum hemorrhage than patients assigned a moderate PPH risk (P < .001).

The number of patients receiving a blood transfusion was 30 (1.3%) in the low PPH group, 12 (2.0%) in the moderate PPH group, and 23 (5.8%) in the high PPH group (P < .001). Patients with a high PPH risk stratification were 4.3 times more likely to receive a blood transfusion than patients in the low PPH risk category. Test characteristics of high-risk stratification for outcome of blood transfusion include a sensitivity of 35.4% (23 of 65 patients who received a blood transfusion identified as high risk), a specificity of 88.2% (not high risk who did not need a blood transfusion), a positive predictive value of 5.8%, and negative predictive value of 98.5%.

## Discussion

The postpartum hemorrhage risk stratification tool implemented at our institution can differentiate between patients at low, moderate, and high risk for postpartum hemorrhage and blood transfusion.

Given this significant difference in transfusion rates and the overall low proportion of patients deemed at high risk for postpartum hemorrhage (12.3%), the benefit of performing a type and cross-match may be considered. The National Partnership for Maternal Safety recommends recognition and prevention of hemorrhage with assessment of hemorrhage risk with every patient at prenatal visits and at time of admission for delivery [10]. However, while there are many risk stratification tools available, they are imperfect. Dilla et al. demonstrated a transfusion rate based on risk assessment of 0.8% for low risk, 2.0% for medium risk, and 7.3% for high risk, which was comparable to our rates [11]. It was argued that a type and screen need only be considered when the risk of transfusion is 5% or higher. While a type and screen usually takes 45 minutes to perform, upgrading to a type and cross with an electronic system only adds 5 minutes. Thus, Dilla et al. suggest having a type and screen for high-risk women, but only type and cross for very high-risk patients when blood needs to be immediately available [11].

While this stratification method is useful in identifying patients at highest risk for postpartum hemorrhage and need for blood transfusion, the plurality of postpartum hemorrhages occurred in the low-risk group (n = 84, 48% of all hemorrhages). The majority of patients who received a transfusion were also in the low-risk group (n = 30, 46% of all patients receiving a transfusion).

In addition to blood preparation, there are other benefits of categorizing patients by risk. Higher risk patients may benefit from preventative measures including higher dose oxytocin or prophylactic tranexamic acid [12]. We have a current ongoing quality improvement initiative which involved higher dose oxytocin with those at high risk for postpartum hemorrhage which has led to a 50% reduction in blood transfusion.

There are limitations to this study. These results may be confounded by the fact that there are statistically significant differences in the cesarean delivery rate between all three groups. The high-risk group was 2.7 times more likely than the low-risk group to undergo a cesarean delivery. The moderate risk group was 2.4 times more likely than the low-risk group to undergo a cesarean delivery. This is likely due to the fact that prior cesarean delivery is a risk factor for postpartum hemorrhage, so all repeat cesarean sections - elective or after failed trial of labor - were categorized as at least moderate risk. Cesarean deliveries in general have higher blood loss than vaginal deliveries, so it is likely that some of the predictive value of this stratification for postpartum hemorrhage comes from its predictive value for cesarean delivery itself [2]. Nevertheless, hemorrhage affects patients regardless of the mode of delivery so it is unclear how this statistical distinction affects the clinical utility of the stratification tool. In addition, the purpose of the risk stratification tool is to assess the patient's postpartum hemorrhage risk upon arrival without knowledge of eventual mode of delivery. Finally, this was an electronic delivery log review. Individual data regarding risks of postpartum hemorrhage were not available.

These findings emphasize that while our stratification method is useful in identifying the highest-risk population, the unpredictable nature of postpartum hemorrhage requires vigilance from all obstetric providers, even in patients deemed to be low risk.

## Conclusions

Risk stratification can differentiate low from high-risk patients for postpartum hemorrhage and associated transfusion or change in hematocrit. The existence of a moderate risk group allows for increased vigilance and establishes a lower threshold to crossing over to high-risk group should risk factors change during labor. However, the majority of patients who receive a transfusion will be classified as low or moderate risk. Thus, all patients should be monitored closely and treated aggressively to prevent morbidity.
